# Return to work after COVID-19: Experiences and expectations from the first wave of COVID-19 in Stockholm

**DOI:** 10.1371/journal.pone.0279000

**Published:** 2022-12-16

**Authors:** Eric Asaba, Lisette Farias, Elisabet Åkesson

**Affiliations:** 1 Department of Neurobiology, Care Science, & Society (NVS), Karolinska Institutet, Stockholm, Sweden; 2 Unit for Research, Education, Development, and Innovation, Stockholms Sjukhem, Stockholm, Sweden; Université de Sherbrooke: Universite de Sherbrooke, CANADA

## Abstract

**Background:**

In Stockholm (Sweden) a substantial number of persons who were infected with SARS-CoV-2 during spring 2020, and received intensive care followed by rehabilitation due to COVID-19, were of working age. For this group, return to work (RTW) is an important part of the rehabilitation, however this is an area that thus far has received little scholarly attention. **The Aim** of this study was two-fold. First, to descriptively look at self-reported work ability over time using the Work Abilty Index among working age adults who recovered from severe COVID-19, and secondly, to explore experiences and expectations concerning RTW among working age adults who recovered from severe COVID-19.

**Methods:**

Focus group interviews and qualitative thematic analyses were utilized. In addition, the study populations’ self-reported work ability index was recorded over one year.

**Findings:**

Qualitative analysis of data resulted in 5 themes: a) Initial experiences after discharge from in-patient rehabilitation, b) Disparate first contact with work, c) Uncertainties about own role in RTW process, d) Working situation for those who had started getting back to work, and e) A need to reprioritize expectations for work in the context of everyday life. There were no statistical differences in work ability index scores between 18 and 52 weeks after discharge from an in-patient rehabilitation unit.

**Conclusion:**

RTW after COVID-19 can require systematic support for several months as well as be initiated earlier in the rehabilitation process. Further research in the area is needed.

## Introduction

Experiences of return to work (RTW) after sick leave due to COVID-19 constitute an area that thus far has received little scientific attention. For working age adults, RTW is an important part of both labour policy as well as the rehabilitation process and maintenance of work after illness or injury resulting in sick leave [1–3]. It has been reported that symptoms can sustain after COVID-19 and include fatigue, decreased physical, psychological, and cognitive function, as well as nutritional problems [4], which can in any combination or a single symptom be challenging to handle without proper support. RTW can further be complicated when the employer lacks knowledge about how to support RTW and when the individual employee is concurrently coping with complex emotions, feelings of uncertainty, anxiety and in some cases posttraumatic stress [5]. A one-size-fits-all solution to RTW, regardless of diagnosis or symptoms, is likely ineffective and thus systematic evaluation and individualized follow-up is important [6]. Although lessons learned from RTW after COVID-19 can contribute to other patient groups with similar difficulties, as with many other conditions, there is a need to look into rehabilitation practices with particular focus on RTW after COVID-19, including both severe COVID-19 at the time of infection and post covid complications [7] appearing after SARS-CoV2 infection and even after initial mild symptoms. This continues to be important because new pandemic waves of COVID-19 driven by novel SARS-CoV2 variants resulting in infections and reinfections with persisting symptoms and secondary complications are repeatedly reported and are expected to continue [4, 8–10]. Moreover, there is a need to build evidence about persons experiences post COVID-19 and the consequences that this has had for work [11]. **The Aim** of this study was two-fold. First, to descriptively look at self-reported work ability over time using the Work Abilty Index among working age adults who recovered from severe COVID-19, and second, to explore experiences and expectations concerning RTW among working age adults who recovered from severe COVID-19.

### Conceptual background

Three concepts are particularly important to define in relation to the aim of this study. First, severe COVID-19 is when an individual´s illness due to SARS-CoV2 infection results in “a SpO_2_ <94% on room air at sea level, a ratio of arterial partial pressure of oxygen to fraction of inspired oxygen (PaO_2_/FiO_2_) <300 mm Hg, a respiratory rate >30 breaths/min, or lung infiltrates >50%” [12].

Second, work ability was originally conceptualized in the 1980’s in the context of aging workers, at the time characterized by “How good is the worker at present, in the near future, and how able is he or she to do his or her work with respect to the work demands, health and mental resources?” [13]. Work ability has over the years been described to include aspects of health resources, competence, values, attitudes, and motivation [13]. Work ability in this context was considered a relevant mechanism to follow up self-reported work ability over time.

Third, RTW is conceptually about a process that has been characterized as "encompassing a series of events, transitions and phases and including interactions with other individuals and the environment" [14]. RTW does in this context therefore not necessarily mean merely an outcome of being in employment but can be about a process. During these transitions and phases, individuals may experience coping with injury or long-term symptoms, being on sick leave, and/or reentering work in a non-linear fashion [14]. Yet, it has been suggested that a significant proportion of people who recovered from COVID-19 have persisting symptoms that can change over time [10], adding to the complexity of RTW and impacting on the need for further studies within the rehabilitation area [15].

### Contextual background

Because RTW differs in different national and international contexts [16], it is important to provide a background to RTW in the context of COVID-19 in Sweden. In the following background, we provide brief information about COVID-19 in Sweden as well as what this has meant for sick leave. We also include a brief background to the law on which sick leave is based.

As of November 14, 2022, over 635 million people globally and 2.62 million people in Sweden had been reported infected by COVID-19 [17]. COVID-19 has led to 6.61 million reported deaths globally and 20,857 reported deaths in Sweden [17]. In Sweden, the greater Stockholm area has had the highest prevalence of COVID-19 with 607,732 reported cases as of November 14, 2022 [17]. The peak number receiving inpatient care for COVID-19 in Stockholm was during April 2020 (1,100 persons). Both the virus infection itself, as well as long term intensive care, may result in multi organ complications calling for strong rehabilitation actions for the patients surviving severe COVID-19 and discharged from ICU [18].

During the period March-September 2020 [19], of the 21,492 persons who were registered on sick leave secondary to COVID-19, 71% were between the ages 18–55 years. The extent of sick leave varied from 15–30 days (55%), 31–45 days (24%), 46–60 days (9%), and more than 60 days (12%). For those who had received intensive care, the median number of days on sick leave was 76 days, and for those who had been hospitalized but not received intensive care, the median number of days on sick leave was 35 days. Moreover, the severity of COVID-19 appears to be predictive of longer sick leave [11]. In Sweden the common term postcovid, as described by The Swedish National Board of Health and Welfare, refers to both remaining symptoms as well as late appearing symptoms after COVID-19 [19]. The cause, prevalence, duration, and prognosis of the prolonged symptoms of COVID-19 are still not clear [20, 21]. Furthermore, healthcare and rehabilitation follow-up provided for persons after COVID-19 has varied, largely because the guidelines for COVID-19 are vague due to a lack of knowledge of the prognosis of the disease [11], and a lack of time to prioritize what and how to follow up aspects of RTW under the COVID-19 pandemic.

It is also relevant to note the legal basis for sick leave because these differ between country contexts. According to Swedish law [22] the possibility to receive compensation for sick leave is based on an assessment of work ability in relation to regular work tasks when the person became ill. Sick leave is typically granted for those who are assessed to not be able to perform work tasks at least 25% of fulltime equivalent. However, the law does not define the concept of work ability [23], which in practice means that the physician who assesses work ability needs to depend on data such as self-reports and assessments from occupational therapy, physical therapy, social work and/or psychology. The assessments for insurance compensation no longer take illness or injury into account, but rather the impact of the illness or injury on work ability. Whereas this assessment of work ability was held in relation to a person’s work and workplace until 2008, the assessment today is held in relation to any type of work in the regular labour market [23]. A consequence of the policy changes of 2008 has been that healthcare providers find it difficult to conduct assessments based on the new guidelines and non-medical administrative officers of the social insurance office find it difficult to assess what level of remuneration to approve based on how medical certificates are formulated. With this as a background, we set out to explore experiences and work ability concerning return to work among working age adults who (at least partially) had recovered from severe COVID-19.

## Methods

### Design

A mixed qualitative exploratory study combining focus group interviews [24, 25] with structured questionnaire data. The Swedish Ethical Review Authority approved the study (dnr: EPM2020-04076).

### Participants and recruitment

A total of 12 persons participated (9 men/3 women) in this study. Participants were informed by (EÅ) about the study after having received inpatient rehabilitation at Stockholms Sjukhem (a private Stockholm based non-profit hospital). The first author (EA) then followed up by phone to answer potential questions about this specific study, go through informed consent, and to book a time for a focus group interview. Follow up telephone contact was made between November 5, 2020 and March 12, 2021, and focus group interviews were carried out between November 13, 2020 –March 22, 2021. A total of 24 persons who had agreed to be contacted received a call by the first author (EA) of which 2 could not be reached. 4 persons did not consent to participate in the study and 2 persons were assessed to not be able to participate due to language difficulties. The remaining 16 persons all agreed to participate, however due to last minute cancellations a total of 12 persons participated in the study. Reasons for last minute cancellations included other commitments and priorities or having forgot about the interview.

All participants had received acute hospitalization with ventilation support at another acute hospital with an intensive care unit prior to in-patient rehabilitation at Stockholms Sjukhem. Inclusion criteria were persons: 1) >18 years of age who gave informed consent, 2) who had SpO_2_ <94% on room air at sea level during hospitalization (according to the NIH-definition), and 3) that worked part or full time before their SARS-CoV-2 infection. Exclusion criteria were: 1) if the person denied informed consent or 2) was not able to communicate sufficiently to actively participate in a focus group interview.

Consent to participate was reconfirmed at the beginning of each focus group interview and each participant returned written consent. Because the number of people who fit the recruitment criteria were relatively small and concentrated to a specific region, demographics of study sample are presented in aggregate to maintain confidentiality. Recruitment for this study was relevant because persons were included who received rehabilitation after a period of intensive care during the 1^st^ wave of COVID-19 spring/summer 2020 were in a unique position to reflect on experiences and expectations about work after COVID-19 during late autumn 2020/early 2021 as well as self-report work ability.

### Demographic and survey data

Each participant was asked to self-report their work ability at 18 and 52 weeks after hospital discharge by filling out the questionnaire “Work Ability Index”, WAI, [26, 27]. The WAI was sent in paper format to all participants prior to the focus group interviews. Each participant was asked to complete and return the WAI. If the WAI had not been returned prior to the focus group interview, participants were asked to complete WAI at the focus group interview site or asked to return the WAI after the focus group interview. All participants also received the WAI by mail as part of a follow-up 1 year after discharge and asked to return the completed form by mail (in a prepaid envelope). Calculation of WAI measures from raw scores based on ten questions covering seven different themes was performed utilizing the publicly available online tool. A WAI score of 7–27 points is viewed as low work ability, 28–36 points as moderate work ability, 37–43 points as good work ability, and 44–49 points as excellent work ability. The WAI is reported to identify early signs of employee unhealth to be able to support actions to contain/ improve work ability.

### Statistical analysis

Demographic data were presented in Table 1 with number of patients (n) and percentages (%) in relation to sex, country of birth, work situation and type of work while descriptive statistics with mean and standard deviation (SD) was reported for continuous values such as age, body mass index, BMI, and number of days of inpatient care. Ordinal variables were reported as median values with an interquartile range (IQR) for hospital anxiety and depression scale (HADS) as well as short physical performance battery (SPPB). The ordinal data from the WAI questionnaire were compared between 18- and 52-weeks post-discharge by using Wilcoxon’s matched pair signed rank test for non-parametric data applying Prism 9.3.0 for MacOs. A p-value <0.05 was referred to as statistically significant.

**Table 1:** Descriptive statistics of severe COVID-19 study sample, 2020.

Category	Variable	RTW Group interview, n = 12 (%)
**Demographic profile**	**Age, years, mean±SD**	55±8.9
	**Male sex, n (%)**	9 (75%)
	**Country of birth**	
	Domestic, n (%)	6 (50%)
	Foreign, n (%)	6 (50%)
	**BMI in specialized rehabilitation, mean±SD**	28.5±5.7
**Work**	Working prior to COVID-19, n (%)	12, full time (100%)
	Back to work 18w after inpatient rehab, n (%)	5, part time (42%)
	Back to work 52 w after inpatient rehab, n (%)	10, ≥part time (83%)
**Type of work**		
	Health care, n (%)	4 (33%)
	Administrative/teacher, n (%)	3 (25%)
	Transportation, n (%)	2 (17%)
	Industrial production, n (%)	2 (17%)
	Social worker, n (%)	1 (8%)
**Inpatient COVID-19 health care**	**Days in total, mean±SD**	55±24
	Days in ICU/ECMO, mean±SD	24±23
	Days in specialized rehabilitation, mean±SD	17±6
**SPPB**	**Total score, at admission to specialized rehab,**	
	In, median (IQR)	5 (3.2)
	**Total Score, at discharge from specialized rehab,**	
	Out, median (IQR)	7.8 (4.2)
**HADS**	**HADS-D, median (IQR) at admission/at discharge**	4 (4.25) / 4(4.75)
	**HADS-A, median (IQR) at admission/at discharge**	3 (2.5) / 3(2.25)

BMI- Body mass index

*ECM- E*xtracorporeal membrane oxygenation

HADS- Hospital anxiety and depression scale (HADS-D refers to the HADS subscale for depression, while HADS-A refers to the subscale for anxiety)

ICU- Intensive care unit

IQR- Interquartile range

SD- Standard deviation

SPPB- Short physical performance battery

### Qualitative data

The focus group interviews were performed approximately 15–20 weeks after discharge from the inpatient rehabilitation, by authors EA and EÅ. The first focus group interview was carried out in one of the meeting rooms at Stockholms Sjukhem (hospital) based on the wishes of the participants and because it was feasible to meet in person between the first and second wave of COVID-19 in Stockholm. The remaining 3 focus group interviews were carried out via a secure online video link system available through the hospital. The change to online focus group interviews was based on requests from some participants and more restrictive recommendations that accompanied the second wave of COVID-19 during the autumn of 2020.

Focus groups interviews were used to raise multiple perspectives about experiences and expectations about RTW. Each session ran 60–90 minutes, and each group consisted of 2–4 participants. It is relevant to note that each session was originally scheduled with 4–5 participants, however last-minute cancellations resulted in fewer participants in each focus group interview. Reasons for cancellation included changes/delays in follow-up times at the rehabilitation clinic, inability to leave work due to last-minute sick leave among co-workers, and feeling ill. To honor the time that other participants had scheduled to meet, decisions were made to run focus group interviews despite the smaller number and instead offer multiple occasions. Focus groups interview questions were explorative and open-ended. Questions were for example: 1) What expectations did you have early in your recovery process about returning to work? 2) How do you think COVID-19 will impact your work in the future? It was possible to probe with questions in order to deepen an understanding of emerging commonalities across experiences or narratives among participants. All sessions were performed by the first and last author, and were audio recorded according to the ethical permit by the National Ethical Review Agency. No video recordings were used. Data were gathered and analyzed by co-authors iteratively.

### Author reflexivity

In keeping with COREQ [28] the following is offered as background to the researchers and reflexivity of how this impacts this study. The first author (EA) has a background in occupational therapy and has worked within the area of return to work within research. He has over 10 years of clinical experience in rehabilitation as an occupational therapist internationally and over 10 years of working with RTW research after neurological diagnoses such as spinal cord injury and stroke. EA had no clinical experience with COVID-19 rehabilitation and no relation to the participants in this study prior to recruitment.

The second author (LF) has a background in occupational therapy and has worked within the area of acute treatment and rehabilitation of adults within neurological clinics in Sweden and abroad. For the past 10 years, she has been involved in qualitative health research and has developed critical reflexivity methods to enhance research processes. LF had no clinical experience with COVID-19 rehabilitation and no relation to the participants in this study prior to recruitment.

The third/last author (EÅ) is an MD/PhD with clinical experience in neurological rehabilitation medicine in Sweden for more than 10 years. She has also conducted experimental and translational neuroscience research in the field of spinal cord injury, stroke and primary neurodegenerative disorders in Sweden and abroad for over 30 years. EÅ has during the COVID-19 pandemic been engaged in the clinical rehabilitation, patient educative programs as well as recruitment of patients as participants in research studies according to ethical permits from The Swedish Ethical Review Authority.

### Qualitative data analysis

In keeping with thematic analytic [24], a back-and-forth process between focus group interviewing and analysis was performed. All audio materials were transcribed verbatim. Although transcripts can be shared with participants upon request, no such requests were made by participants in this study. These transcripts were carefully read and coded in three steps. All materials were analyzed in the original language (Swedish), and quotations used in this article were translated to English by the first and second author and compared for accuracy. The data was analyzed inductively and although this process involved overlapping between steps, the procedure is outlined stepwise here for the purposes of clarity. All data were independently coded by the first (EA) and second author (LF). The authors met online to compare and discuss the analysis. The authors considered that data saturation was reached when similar experiences and expectations were iterated during focus group interviews. All authors were part of a discussion and interpretations of the findings.

In the first step, open coding entailed identifying and breaking the text into manageable bits that were assigned codes to capture the essence of data in a few words. Examples of early codes were: *energy is worse*, *far from well*, *routines for returning to work*. In the second step, codes were merged, and previously coded transcripts were revisited. Existing, as well as new codes within and across data were utilized in a back and forth process, which allowed for both open and focused coding. Codes such as *routines for returning to work* were merged and codes such as *working out a plan together* and *follow-up*, which contributed to abstraction of the data. In the third step, similar codes were consolidated and organized into coherent groupings whereby 5 themes were identified. These themes represent experiences and expectations concerning RTW after severe COVID-19.

## Results

### Work ability index

**Fig 1:**
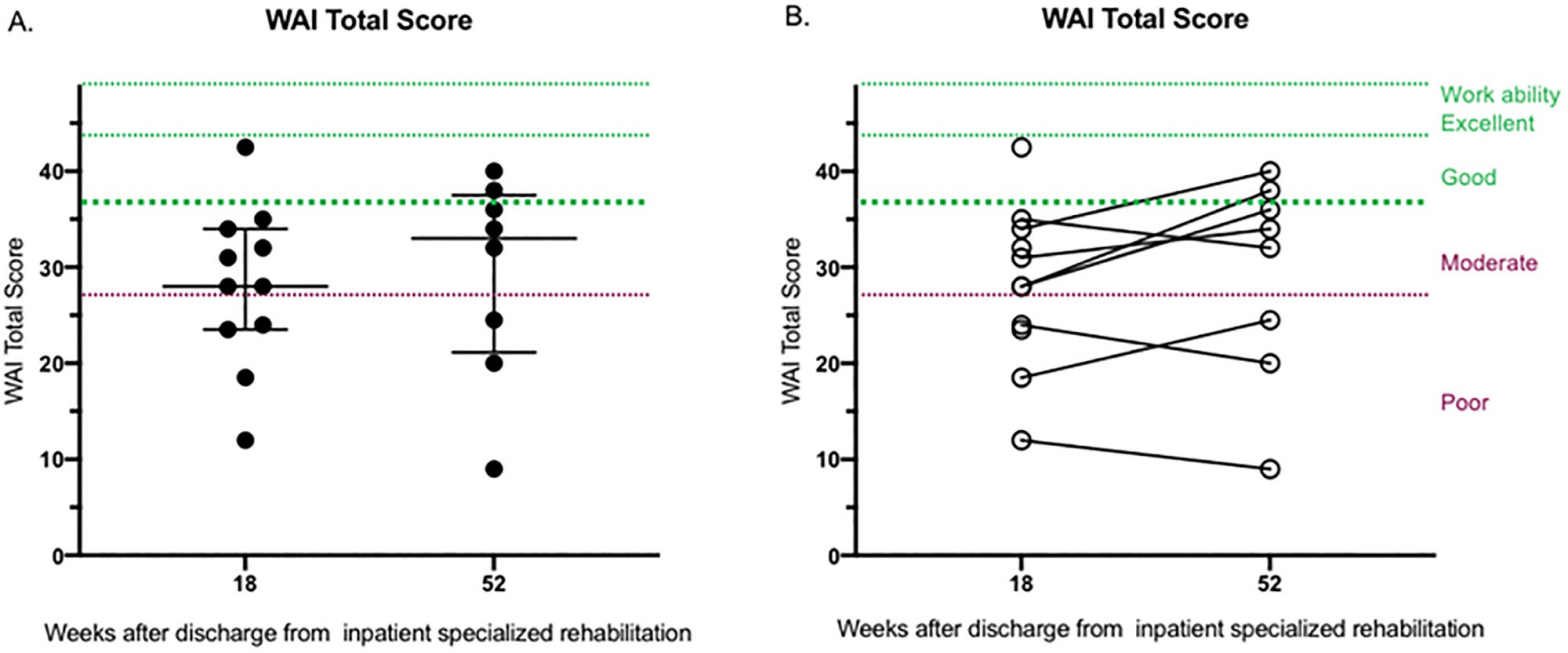
WAI total score per person at 18 and 52 weeks (A) and WAI total score change per person from 18 to 52 weeks (B) after inpatient rehabilitation discharge. The level of work ability according to WAI score is indicated by dotted lines. There was no statistically significant difference in the WAI score between 18 and 52 weeks post discharge, p = 0.19.

The WAI scores for the participants in this study are reported in Table 2 and Fig 1.

**Table 2:** Work ability after severe COVID-19.

**Work Ability Index**	**Total score, max 49**
	18 weeks after discharge from inpatient rehab, median (IQR): 28 (10.5)
	52 weeks after discharge from inpatient rehab, median (IQR): 33 (16.4)
**Work Ability Score**	**Q1 score, max 10**
	18 weeks after discharge from inpatient rehab, median (IQR): 6 (2)
	52 weeks after discharge from inpatient rehab, median (IQR): 6.5 (3.5)

IQR- Interquartile range

Q1- Question 1 in the Work Ability Index (WAI) questionnaire

At 18 weeks after discharge from the inpatient rehabilitation clinic, the participants self-reported a median WAI score of 28 (n = 11, with an interquartile range of 10.5, with a minimum of 12 up to a maximal score of 42.5 among the participants). At 52 weeks after discharge, the median WAI score was instead 33 (n = 8, with an interquartile range of 16.4, with a minimum of 9 up to a maximal score of 40 among the participants). There was no statistically significant difference in the WAI score from 18 to 52 weeks after discharge from the inpatient rehabilitation clinic, p = 0.19 in this study population. Three participants lowered their total WAI score at 52 weeks compared to 18 weeks after discharge. The work contexts for participants in the study mirrored the broader picture in Stockholm. Nearly half of the participants in this study worked within the healthcare sector and had been exposed for SARS-CoV2 at work. Participants who worked as teachers and within transportation, also represented among participants in this study, were also in an exposed group who were unable to work from home during the initial stages of the pandemic.

### Focus group interview

Qualitative findings from the analysis are presented in five themes. Although the naming of each theme is intended to be representative, each theme encompasses more than the phrase: a) Initial experiences after discharge from in-patient rehabilitation, b) Disparate first contact with work, c) Uncertainties about own role in RTW process, d) Working situation for those who had started getting back to work, and e) A need to reprioritize expectations for work in the context of everyday life. The participants interviewed in this study unanimously communicated an interest in returning to work, although some participants also felt that early retirement would be preferable. Pseudonyms have been used for ease of read in the presentations of quotes.

### Initial experiences after discharge from in-patient rehabilitation

Findings showed that a sense of immediate disruption in everyday life was not limited to first becoming ill, but also central to the narratives about everyday life including work after discharge from in-patient rehabilitation. The focus on physically regaining strength was explicit, however, the need for other aspects in the recovery process was also recognizable.

When one was discharged one was discharged from healthcare, and then nobody follows up how one was actually, one’s thoughts, because I felt like, I have never been so sick in my entire life. So I actually thought that I was going to die, and there was nobody that talked about that. So it has actually mostly been a focus on this training stuff (Victoria).

Early experiences after discharge gave space to consider the consequences of having been acutely ill. It also provided, as participants put it, time to reconsider personal priorities in relation to both private and working life.

First of all, I am not going to do something in the future like this, started thinking about that. Right now I live in the moment. And at work, it is going to be the same. All that I manage, that’s ok. I am not going to stress myself or force my body to do anything (Kirstin).

Finally, experiences after discharge that sustained were the sentiments of prolonged fatigue and several other symptoms that persisted many months after discharge from the hospital.

So I don’t know, what should one do to come back to work? It depends on what you do of course. But I think it has gotten better. It is slow. I don’t think that anyone likes to be ill for this long or have problems, because I also have those…besides the energy, I walk quite a lot. And when it is plane it is ok, when I walk uphill it is 100 times worse (Mohammad).

These persistent symptoms in combination with a lack of follow-up were described by participants as a problem that affected their confidence about when to re-enter working life and how to manage other aspects of everyday life.

### Disparate first contact with work

Experiences of the initial contacts with work after regaining consciousness varied among participants. One person had terminated employment, others described only scarce communication, and some had more concrete and regular contact with their immediate managers. One participant described the shock of losing his job:

My employer has not had any contact with me at all, but he has had communication with my partner, and all those weeks and months that passed he has not mentioned one word about that I was one of the employees that were let go (Matthew).

Another participant described scarce communication albeit he had received support from healthcare providers. He said:

No nothing through my employer. But I go to a bunch of, I mean like, a physical therapist and social worker and like that. But nothing from my employer (Mohammad).

A participant who had regular contact with the employer shared:

Yes, but my employer or manager, she has called me regularly. It was she that nagged me to go to the hospital too. I was trying to be so good and I thought it would pass after a couple of weeks at home, but it did not happen. And she calls me once to twice a week. Has done so throughout my entire sick leave (Victoria).

The initial contacts as well as what has for some turned out to be regular contact varied across participants. The experiences shared demonstrate certain inconsistencies about the degree to which dialogue about RTW was initiated and maintained with an employer.

### Uncertainties about own role in RTW process

Participants shared experiences that indicated that they did not always know what policies existed in terms of sick leave and RTW but showed understanding of the economic consequences of short-term as well as extended sick leave. Most participants knew that they had insurance that covered a certain number of days of sick leave and that they needed to submit documentation in order to receive benefits. Most participants also expected that people would consider or understand that their transitioning from a state of complete sick leave to being back at work required time and energy that was experienced as challenging.

But just this situation, here one should also take into consideration or realize in some way that here there are many that don’t have the energy to be active. It is part of the illness. So I think that those who are decision makers should be able to have more empathy to have an understanding of this special (not ordinary) illness actually. (Sven)

Participants experienced that they were expected to coordinate dialogue between their employer and healthcare. This was by many perceived as challenging.

My employer has also asked me if we could meet together with my doctor, we three. I have talked to my doctor, but the doctor says no. I asked him that the social insurance office had called me once, and my job, and that they wanted to know how they can do better for me so that I can come back to work (Kirstin).

Another participant also raised a need for focused professional support that specifically focused on support after the acute phase.

If one could wish at least, a team that could focus more specifically on those that have suffered from this pandemic and coordinate care of the problems that have specifically remained after one has recovered from the acute phase (Alex).

Participants also experienced that it would have been helpful if there was a natural communication between healthcare providers and employers with regard to RTW. This way, the expectations of initiating or coordinating a dialogue about RTW would not need to be on the individual, which risks missing important aspects.

My thought is that, healthcare then, when one has been hospitalized like we have for a long time and so on, they have good knowledge about us. They should be able to provide the employers with some knowledge, or some link should be available here, whoever it might be, a contact with the employer that says, ‘you have an employee that has experienced this/that. We just want to ask, do you have a plan for return to work, and do you need any help from us?’ (Sven)

### Working situation for those who had started getting back to work

RTW after COVID-19 was also about re-evaluating priorities and expectations concerning work and private life. For participants who had returned to work, this meant a shift in focus from recovering/surviving to slowly building up strength to manage work tasks and everyday life. Participants shared experiences about adaptations at work to compensate for a lack of energy although this was not equally successful for everyone. Mohammad said,

Yes, I work. As usual basically, but I manage a bit worse, so it is a bit slower pace and things like that. Unfortunately, I compensate for this with longer working days, so it is not successful at all.

For others, it had to do with slowing down expectations about work by situating it as one of several priorities. Feeling that fulltime work was not yet possible, was not only a matter of symptoms that remained after COVID-19, but also about time for other commitments such as childcare and parenting. One participant said,

I feel that I have a long way to go before I am back to normal working life again, as I see it. I mean I don’t want to go to a new job and still have pain in my body, that I don’t want. So yes, I am trying to make things work in everyday life…I have four kids also that, well take care of them also. It’s not only about me. There needs to be energy for that as well, and that I feel I don’t have. Not now at least. (Matthew)

Other perspectives also were raised concerning sentiments of having returned too early. One participant who was in the 30’s had recovered relatively quickly after intensive care and rehabilitation.

I cannot react on, like just as quickly as I had wished, or act on a situation if something happens in the building, if I say so. Or protect if something happens to me or a patient. But that will, we’ll see later actually how that develops. I mean purely cognitively it is not a problem to return to work. I have been very active. No problem to keep my concentration. But I am not fully myself in my job (Andrew).

The participant had spoken with the employer and returned to work without any rehabilitation plan or transition to increase time at work. In retrospect, the participant raises certain challenges that have become more evident.

### A need to reprioritize expectations for work in the context of everyday life

Participants expressed an expectation for RTW that later turned out to not match what they were able to do. Factors such as concentration, memory, and physical endurance were examples of challenges that impacted on their ability to perform at work, which led to a need to change what to expect in order to achieve a sense of satisfaction in everyday life.

I am used to being up around 22,000 and 25,000 steps per day, and I do not do that now. And then this concentration difficulty. And then I was shocked when I realized I had difficulty writing. (Victoria)

Another participant said,

For my job, I still have things that I cannot handle. It is one of the things that matter most, it is my memory. It is memory and concentration, that one that is in my head for example. In the body, there are lots. (Kirstin)

Moreover, participants received rehabilitation services and support that were primarily focused on the physical aspects of endurance and strength. However, participants shared a need to receive services to support a broader spectrum of aspects related to everyday life including work.

And there are different like, pulmonologist, cardiologist, and all sorts. But what I experience there again then, it is that question about. One focuses, so to speak, on the physical rehabilitation a lot. And partly on cognition, but not directly connected to work ability. I think that it feels like they should do a better analysis in the follow-up connected to work ability (Sven).

Due to regulations for rehabilitation, rehabilitation services were sometimes terminated when the person began working, which was for some participants unexpected. They shared that they expected to receive rehabilitation services for a longer period and in the absence of continued rehabilitation services the RTW process felt more challenging.

I thought it was very good with rehab training. And that I only had that as long as I was full time on sick leave but as soon as I started working 50%, it stopped. And I think I probably have had enough but I would have benefitted from a bit more, even when I started working a bit, because it was very good. (Alex)

There was a clear expression of need for support that included a broad spectrum of areas such as physical endurance, memory, work, and ways to make it fit in everyday life. Moreover, participants in this study raised the importance of the change in needs and expectations over time, and that they did not understand what needs they would have once they began working.

## Discussion

The aim of this study was to descriptively look at self-reported work ability and to explore experiences and expectations concerning RTW among working age adults who recovered from severe COVID-19. The WAI is reported of value to identify early signs of employee unhealth with the aim to be able to support actions to maintain or improve work ability [26, 29]. In the context of the COVID-19 pandemic, WAI has been used in several studies where focus has been on work ability among workers based on changes in the work environment [30, 31]. In this study we found it relevant to use the WAI for persons who were recovering from COVID-19 because the instrument is easy to complete and provides a good sense of self-reported work ability. A work ability of at least 37 is considered necessary in relation to being able to sustain present work [27]. Only 2 of the study participants reported a work ability score above 37, the threshold for good work ability, despite a majority of the group members already having initiated work at least part time. These results follow a similar trend as what has been descriptively reported in a study from Italy [32]. In the present study, no significant difference in work ability was reported at 52 compared to 18 weeks after hospital discharge at the group level, meaning that symptoms post COVID-19 persist longer than expected, which could be indicative that RTW may require follow-up of persistent and changing symptoms as well as flexible adaptation of work tasks. However, there was great heterogeneity in the self-reported work ability. The heterogeneity in self-reported work ability can have reflected the change in complications and symptoms over time after severe COVID-19, which can be indicative of a need for individualized long-term rehabilitation plans and interventions. Although the relative number of persons that are severely impacted by symptoms after COVID-19 might be low, there does appear to be evidence for that it is more challenging to return to work after a hospitalization for severe COVID-19 [33].

Moreover, the qualitative findings from this study raise some important topics for consideration. Experiences among participants in this project mirror much of what has been found elsewhere [34]. In this study, people who had returned to work after COVID-19 anticipated no problems, but later realized that they were unable to perform their work tasks as before or that they were overly exhausted from balancing work and private life. These experiences were also supported by the WAI data. It can be relevant to consider the need for supporting people to negotiate expectations about RTW and work ability based on personally reported experiences and WAI results. It can also be important that a wide array of aspects of work ability, and not only physical performance, is evaluated.

Despite that there are laws that regulate sick leave and rehabilitation processes to support RTW, there is a lack of consistency and systematic methods applied with populations recovering from COVID-19. Depending on manager, employer, employer policies, and type of work, to name some factors, the structural conditions for initiating a dialogue about RTW appear to vary greatly. Participants shared experiences that were at times inconsistent, i.e., information received about prognosis, a dialogue about future horizons of recovery, and how consequences of COVID-19 could impact RTW. Although there was an understanding that this was a new disease, and that information about prognosis or recovery was understandably challenging, consequences of intensive care and extended sick leave have been known even before the COVID-19 pandemic. Moreover, based on findings from prior research an early but timely dialogue between employer, employee and rehabilitation staff regarding work ability and actual work tasks would be important [1, 35].

The participants also expressed a need and expectation for more focused support. Because much of the responsibility of coordinating RTW is on the individual who is on sick leave, it can be difficult to know what is needed unless the person happens to have this knowledge from other prior experiences. This dilemma is known from other studies in which RTW has been explored from the perspective of i.e. persons with stroke [36–38], spinal cord injury [1, 35, 39], cancer [40, 41], and mental illness [42]. Common for all these groups in a Swedish context, has been the challenge of coordinating the different health system and insurance requirements as well as receiving timely and relevant support. Moreover, challenges regarding work have been found to change over time for the individual. A practical implication of this study is that, when planning RTW for all these groups, authorities should provide support for coordinating the different health efforts and systems that are required. This means that a coordinator or health professional that could conduct follow-ups and coordination meetings is needed as argued in other Swedish RTW studies [1, 39, 42]. Another implication is that employers need to have a more comprehensive understanding of this group, since this study illustrates that experienced challenges related to work can be individual and can change over time.

### Strengths and limitations of the study

There are several limitations to this study. It was not possible to conduct data gathering onsite for all focus group interviews due to pandemic restrictions. On the other hand, it was not our assessment that the online focus group format diminished the data gathering process. This is in keeping with recent reports that online audio-visual focus groups have been found to be acceptable for participants and can be easily accessible, time-effective, facilitate engagement with others, contribute to rapport, and provide a relevant way to exchange experiences [43]. The data was gathered during the end of the first wave and beginning of second wave, when the burden of being asked to participate in studies was high for all involved, which can have contributed to missing follow-up data with regard to WAI from three participants. The participants in this study received initial acute and intensive care at any of the hospitals in the Stockholm area and were then later referred to Stockholms Sjukhem for inpatient rehabilitation where recruitment for this study was carried out.

According to the national quality registry for rehabilitation, WebRehab, during the 1^st^ wave 57% of persons with confirmed COVID-19 who received inpatient rehabilitation after severe COVID-19 were born outside of Sweden. Nationally, a clear majority were male (75%) and the mean age was 55 years of age. During the 2^nd^ wave, 37% of the confirmed cases who received inpatient rehabilitation after severe COVID-19 were born outsides of Sweden. Certain professions such as taxi and bus drivers were reported to have a 4.8 higher incidence rate compared to other professions in Sweden during the spring of 2020, professions that also were represented among participants in the present study [44]. The participants included in this study appear to represent a similar demographic, which can be seen as a strength.

## Conclusion

Participants in this study had postcovid complications associated with COVID-19 that persisted one year after discharge from in-patient rehabilitation, and several participants had not yet returned to work full-time after one year. Participants who had returned to work in this study, initially expected work to be possible and later realized that certain work tasks were difficult to perform as before. There is a need for interprofessional, structured, and longer follow-up as well as coordination of rehabilitation services. These services should focus on RTW situated in everyday life, engaging the person on sick leave in goal setting and a future horizon of what can be possible.
